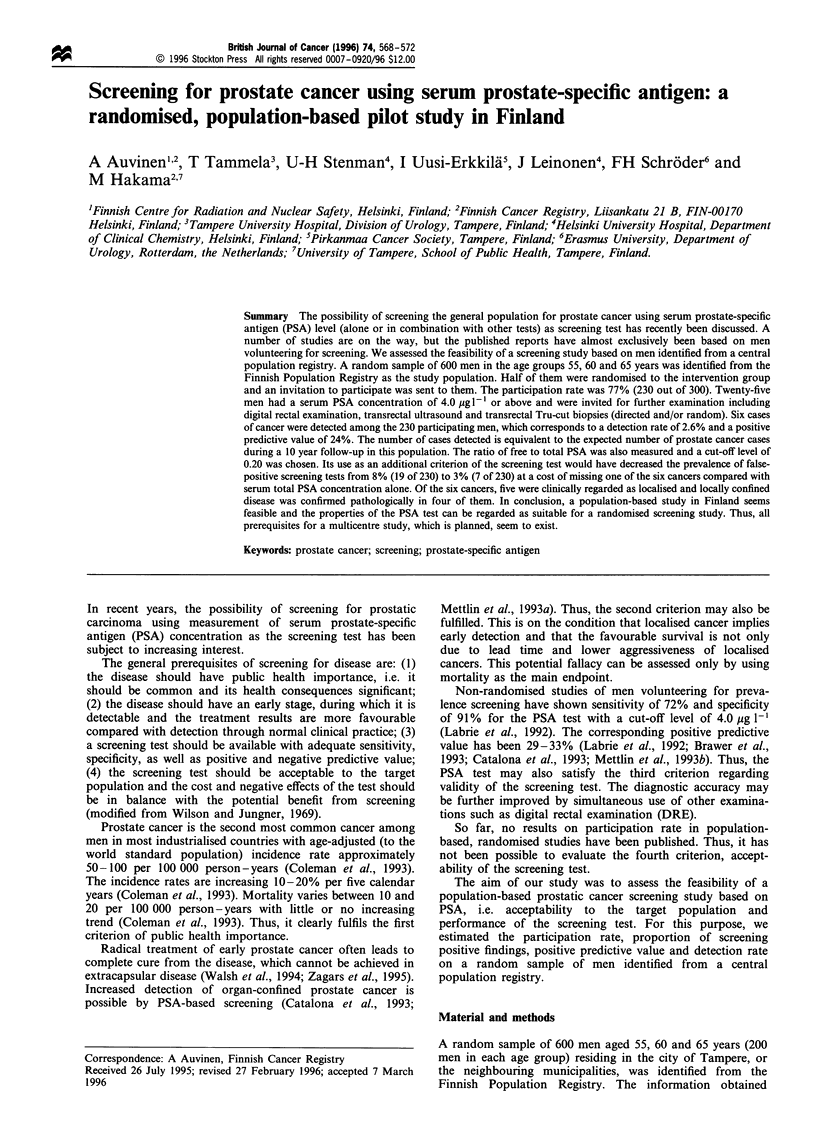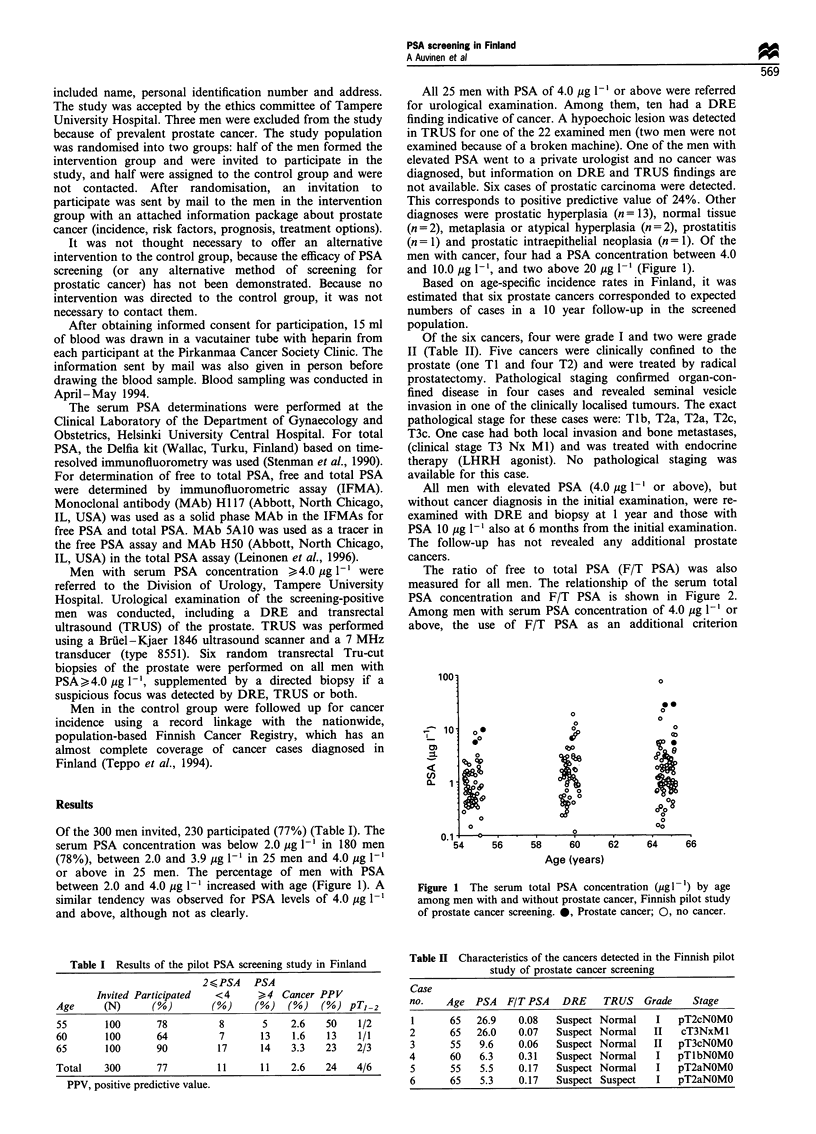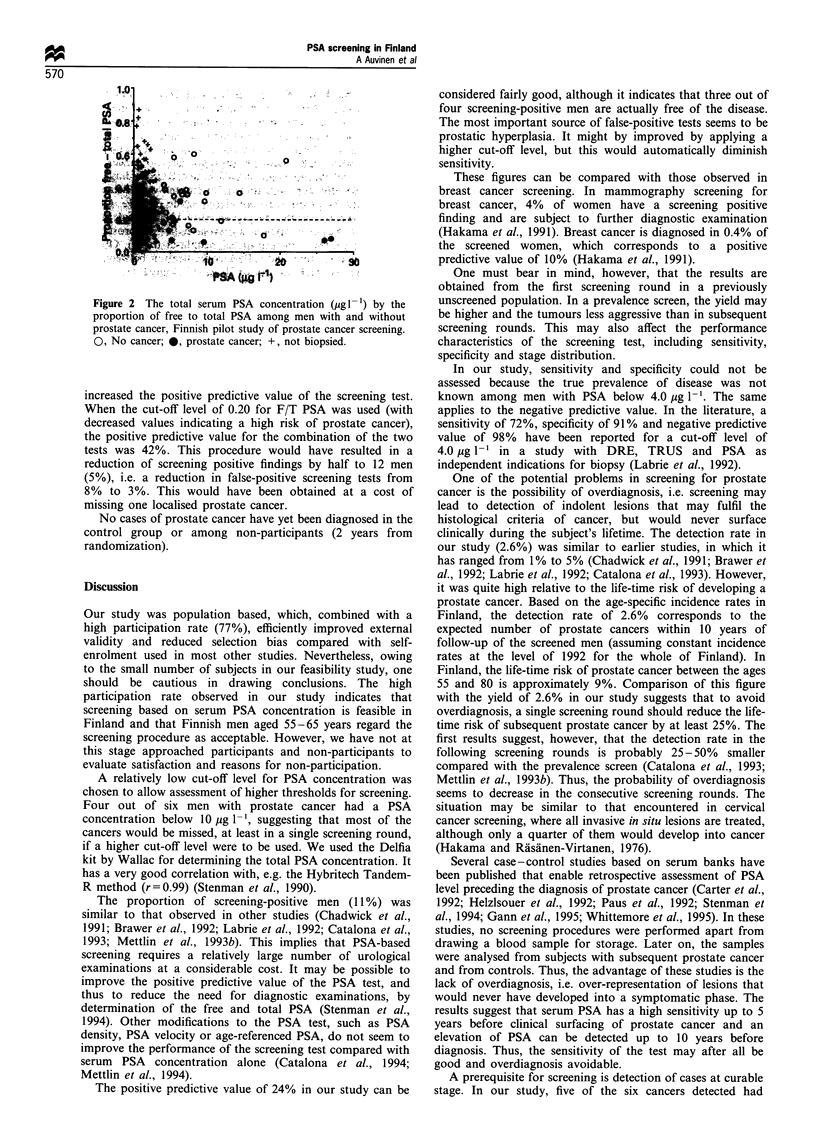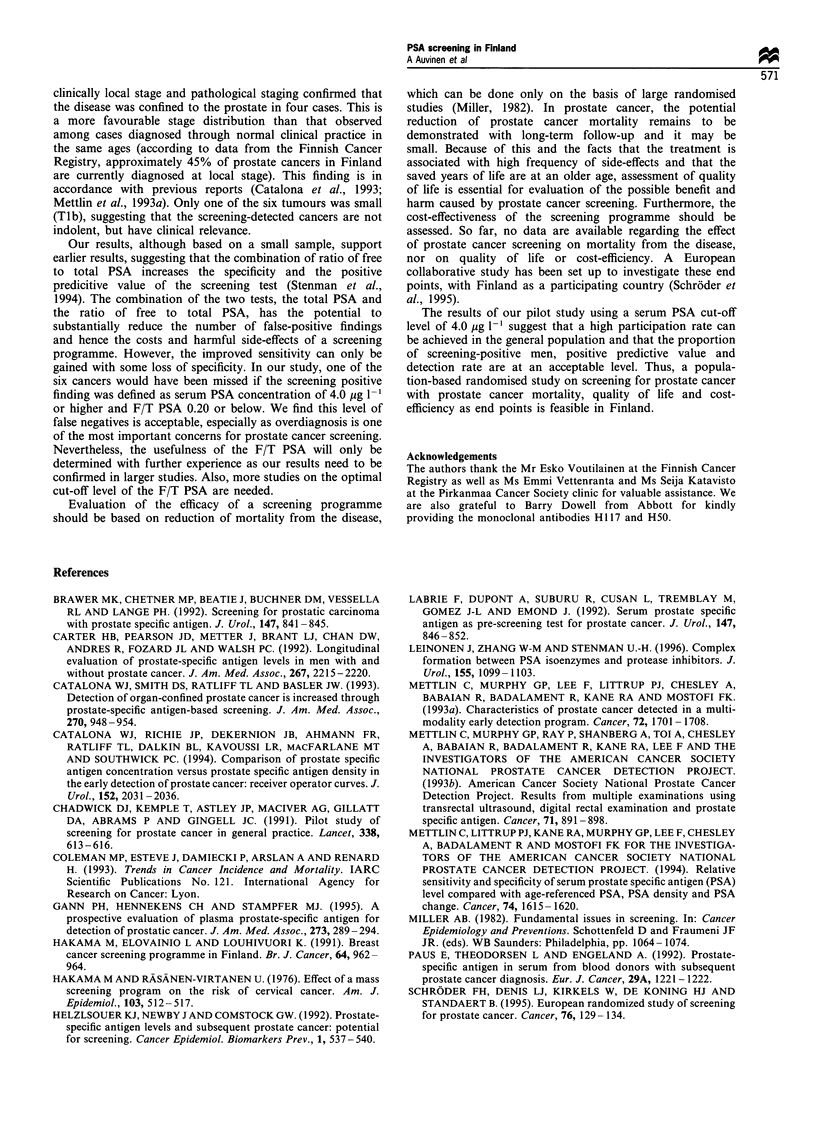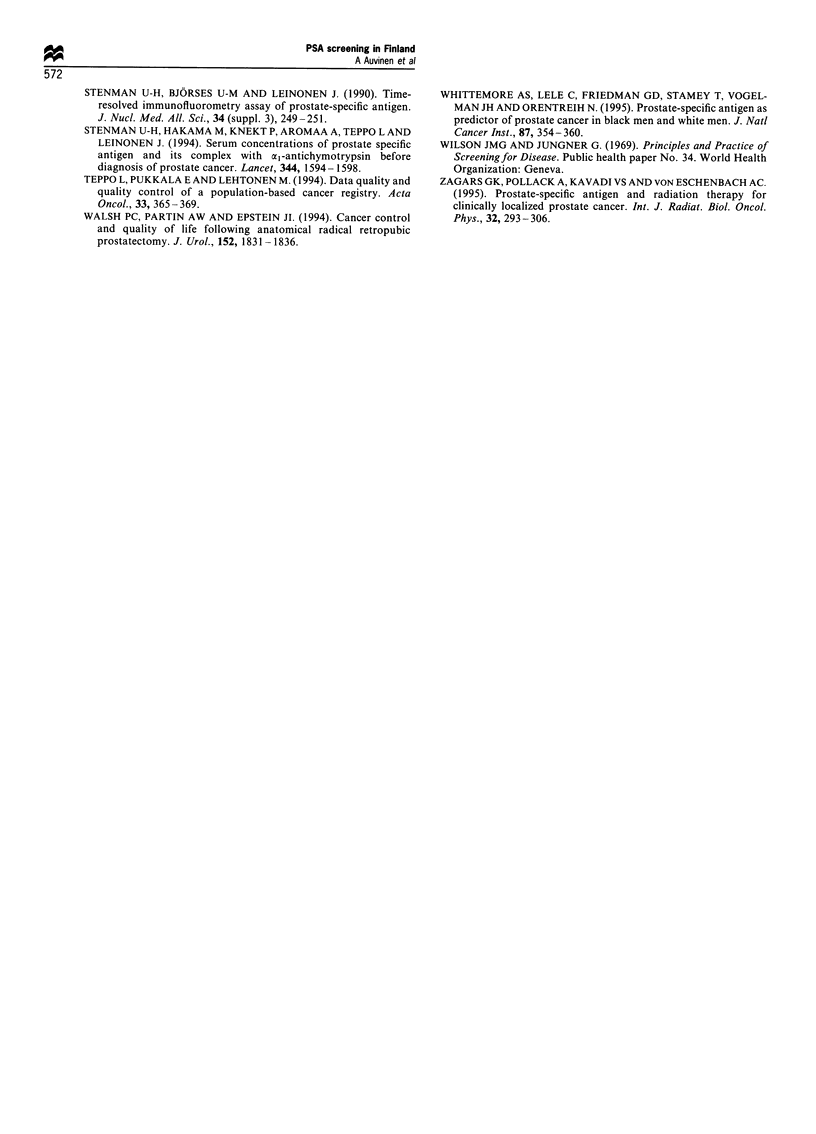# Screening for prostate cancer using serum prostate-specific antigen: a randomised, population-based pilot study in Finland.

**DOI:** 10.1038/bjc.1996.402

**Published:** 1996-08

**Authors:** A. Auvinen, T. Tammela, U. H. Stenman, I. Uusi-Erkkilä, J. Leinonen, F. H. Schröder, M. Hakama

**Affiliations:** Finnish Centre for Radiation and Nuclear Safety, Helsinki, Finland.

## Abstract

The possibility of screening the general population for prostate cancer using serum prostate-specific antigen (PSA) level (alone or in combination with other tests) as screening test has recently been discussed. A number of studies are on the way, but the published reports have almost exclusively been based on men volunteering for screening. We assessed the feasibility of a screening study based on men identified from a central population registry. A random sample of 600 men in the age groups 55, 60 and 65 years was identified from the Finnish Population Registry as the study population. Half of them were randomised to the intervention group and an invitation to participate was sent to them. The participation rate was 77% (230 out of 300). Twenty-five men had a serum PSA concentration of 4.0 micrograms l-1 or above and were invited for further examination including digital rectal examination, transrectal ultrasound and transrectal Tru-cut biopsies (directed and/or random). Six cases of cancer were detected among the 230 participating men, which corresponds to a detection rate of 2.6% and a positive predictive value of 24%. The number of cases detected is equivalent to the expected number of prostate cancer cases during a 10 year follow-up in this population. The ratio of free to total PSA was also measured and a cut-off level of 0.20 was chosen. Its use as an additional criterion of the screening test would have decreased the prevalence of false-positive screening tests from 8% (19 of 230) to 3% (7 of 230) at a cost of missing one of the six cancers compared with serum total PSA concentration alone. Of the six cancers, five were clinically regarded as localised and locally confined disease was confirmed pathologically in four of them. In conclusion, a population-based study in Finland seems feasible and the properties of the PSA test can be regarded as suitable for a randomised screening study. Thus, all prerequisites for a multicentre study, which is planned, seem to exist.


British Journal of Cancer (1996) 74, 568-572
© 1996 Stockton Press All rights reserved 0007-0920/96 $12.00

### Screening for prostate cancer using serum prostate-specific antigen: a randomised, population-based pilot study in Finland

A Auvinen[1,2], T Tammela[3], U-H Stenman[4], I Uusi-Erkkilä[5], J Leinonen[4], FH Schröder[6] and M Hakama[2,7]

[1]*Finnish Centre for Radiation and Nuclear Safety, Helsinki, Finland;* [2]*Finnish Cancer Registry, Liisankatu 21 B, FIN-00170 Helsinki, Finland;* [3]*Tampere University Hospital, Division of Urology, Tampere, Finland;* [4]*Helsinki University Hospital, Department of Clinical Chemistry, Helsinki, Finland;* [5]*Pirkanmaa Cancer Society, Tampere, Finland;* [6]*Erasmus University, Department of Urology, Rotterdam, the Netherlands;* [7]*University of Tampere, School of Public Health, Tampere, Finland.*

**Summary** The possibility of screening the general population for prostate cancer using serum prostate-specific antigen (PSA) level (alone or in combination with other tests) as screening test has recently been discussed. A number of studies are on the way, but the published reports have almost exclusively been based on men volunteering for screening. We assessed the feasibility of a screening study based on men identified from a central population registry. A random sample of 600 men in the age groups 55, 60 and 65 years was identified from the Finnish Population Registry as the study population. Half of them were randomised to the intervention group and an invitation to participate was sent to them. The participation rate was 77% (230 out of 300). Twenty-five men had a serum PSA concentration of 4.0 $\mu g l^{-1}$ or above and were invited for further examination including digital rectal examination, transrectal ultrasound and transrectal Tru-cut biopsies (directed and/or random). Six cases of cancer were detected among the 230 participating men, which corresponds to a detection rate of 2.6% and a positive predictive value of 24%. The number of cases detected is equivalent to the expected number of prostate cancer cases during a 10 year follow-up in this population. The ratio of free to total PSA was also measured and a cut-off level of 0.20 was chosen. Its use as an additional criterion of the screening test would have decreased the prevalence of false-positive screening tests from 8% (19 of 230) to 3% (7 of 230) at a cost of missing one of the six cancers compared with serum total PSA concentration alone. Of the six cancers, five were clinically regarded as localised and locally confined disease was confirmed pathologically in four of them. In conclusion, a population-based study in Finland seems feasible and the properties of the PSA test can be regarded as suitable for a randomised screening study. Thus, all prerequisites for a multicentre study, which is planned, seem to exist.

**Keywords:** prostate cancer; screening; prostate-specific antigen

In recent years, the possibility of screening for prostatic carcinoma using measurement of serum prostate-specific antigen (PSA) concentration as the screening test has been subject to increasing interest.

The general prerequisites of screening for disease are: (1) the disease should have public health importance, i.e. it should be common and its health consequences significant; (2) the disease should have an early stage, during which it is detectable and the treatment results are more favourable compared with detection through normal clinical practice; (3) a screening test should be available with adequate sensitivity, specificity, as well as positive and negative predictive value; (4) the screening test should be acceptable to the target population and the cost and negative effects of the test should be in balance with the potential benefit from screening (modified from Wilson and Jungner, 1969).

Prostate cancer is the second most common cancer among men in most industrialised countries with age-adjusted (to the world standard population) incidence rate approximately 50-100 per 100 000 person-years (Coleman *et al.*, 1993). The incidence rates are increasing 10-20% per five calendar years (Coleman *et al.*, 1993). Mortality varies between 10 and 20 per 100 000 person-years with little or no increasing trend (Coleman *et al.*, 1993). Thus, it clearly fulfils the first criterion of public health importance.

Radical treatment of early prostate cancer often leads to complete cure from the disease, which cannot be achieved in extracapsular disease (Walsh *et al.*, 1994; Zagars *et al.*, 1995). Increased detection of organ-confined prostate cancer is possible by PSA-based screening (Catalona *et al.*, 1993; Mettlin *et al.*, 1993*a*). Thus, the second criterion may also be fulfilled. This is on the condition that localised cancer implies early detection and that the favourable survival is not only due to lead time and lower aggressiveness of localised cancers. This potential fallacy can be assessed only by using mortality as the main endpoint.

Non-randomised studies of men volunteering for prevalence screening have shown sensitivity of 72% and specificity of 91% for the PSA test with a cut-off level of 4.0 $\mu g l^{-1}$ (Labrie *et al.*, 1992). The corresponding positive predictive value has been 29-33% (Labrie *et al.*, 1992; Brawer *et al.*, 1993; Catalona *et al.*, 1993; Mettlin *et al.*, 1993*b*). Thus, the PSA test may also satisfy the third criterion regarding validity of the screening test. The diagnostic accuracy may be further improved by simultaneous use of other examinations such as digital rectal examination (DRE).

So far, no results on participation rate in population-based, randomised studies have been published. Thus, it has not been possible to evaluate the fourth criterion, acceptability of the screening test.

The aim of our study was to assess the feasibility of a population-based prostatic cancer screening study based on PSA, i.e. acceptability to the target population and performance of the screening test. For this purpose, we estimated the participation rate, proportion of screening positive findings, positive predictive value and detection rate on a random sample of men identified from a central population registry.

### Material and methods

A random sample of 600 men aged 55, 60 and 65 years (200 men in each age group) residing in the city of Tampere, or the neighbouring municipalities, was identified from the Finnish Population Registry. The information obtained

Correspondence: A Auvinen, Finnish Cancer Registry
Received 26 July 1995; revised 27 February 1996; accepted 7 March 1996

included name, personal identification number and address. The study was accepted by the ethics committee of Tampere University Hospital. Three men were excluded from the study because of prevalent prostate cancer. The study population was randomised into two groups: half of the men formed the intervention group and were invited to participate in the study, and half were assigned to the control group and were not contacted. After randomisation, an invitation to participate was sent by mail to the men in the intervention group with an attached information package about prostate cancer (incidence, risk factors, prognosis, treatment options).

It was not thought necessary to offer an alternative intervention to the control group, because the efficacy of PSA screening (or any alternative method of screening for prostatic cancer) has not been demonstrated. Because no intervention was directed to the control group, it was not necessary to contact them.

After obtaining informed consent for participation, 15 ml of blood was drawn in a vacutainer tube with heparin from each participant at the Pirkanmaa Cancer Society Clinic. The information sent by mail was also given in person before drawing the blood sample. Blood sampling was conducted in April–May 1994.

The serum PSA determinations were performed at the Clinical Laboratory of the Department of Gynaecology and Obstetrics, Helsinki University Central Hospital. For total PSA, the Delfia kit (Wallac, Turku, Finland) based on time-resolved immunofluorometry was used (Stenman *et al.*, 1990). For determination of free to total PSA, free and total PSA were determined by immunofluorometric assay (IFMA). Monoclonal antibody (MAb) H117 (Abbott, North Chicago, IL, USA) was used as a solid phase MAb in the IFMAs for free PSA and total PSA. MAb 5A10 was used as a tracer in the free PSA assay and MAb H50 (Abbott, North Chicago, IL, USA) in the total PSA assay (Leinonen *et al.*, 1996).

Men with serum PSA concentration $\geqslant 4.0\ \mu g\ l^{-1}$ were referred to the Division of Urology, Tampere University Hospital. Urological examination of the screening-positive men was conducted, including a DRE and transrectal ultrasound (TRUS) of the prostate. TRUS was performed using a Brüel–Kjaer 1846 ultrasound scanner and a 7 MHz transducer (type 8551). Six random transrectal Tru-cut biopsies of the prostate were performed on all men with PSA$\geqslant 4.0\ \mu g\ l^{-1}$, supplemented by a directed biopsy if a suspicious focus was detected by DRE, TRUS or both.

Men in the control group were followed up for cancer incidence using a record linkage with the nationwide, population-based Finnish Cancer Registry, which has an almost complete coverage of cancer cases diagnosed in Finland (Teppo *et al.*, 1994).

### Results

Of the 300 men invited, 230 participated (77%) (Table I). The serum PSA concentration was below 2.0 $\mu g\ l^{-1}$ in 180 men (78%), between 2.0 and 3.9 $\mu g\ l^{-1}$ in 25 men and 4.0 $\mu g\ l^{-1}$ or above in 25 men. The percentage of men with PSA between 2.0 and 4.0 $\mu g\ l^{-1}$ increased with age (Figure 1). A similar tendency was observed for PSA levels of 4.0 $\mu g\ l^{-1}$ and above, although not as clearly.

All 25 men with PSA of 4.0 $\mu g\ l^{-1}$ or above were referred for urological examination. Among them, ten had a DRE finding indicative of cancer. A hypoechoic lesion was detected in TRUS for one of the 22 examined men (two men were not examined because of a broken machine). One of the men with elevated PSA went to a private urologist and no cancer was diagnosed, but information on DRE and TRUS findings are not available. Six cases of prostatic carcinoma were detected. This corresponds to positive predictive value of 24%. Other diagnoses were prostatic hyperplasia ($n=13$), normal tissue ($n=2$), metaplasia or atypical hyperplasia ($n=2$), prostatitis ($n=1$) and prostatic intraepithelial neoplasia ($n=1$). Of the men with cancer, four had a PSA concentration between 4.0 and 10.0 $\mu g\ l^{-1}$, and two above 20 $\mu g\ l^{-1}$ (Figure 1).

Based on age-specific incidence rates in Finland, it was estimated that six prostate cancers corresponded to expected numbers of cases in a 10 year follow-up in the screened population.

Of the six cancers, four were grade I and two were grade II (Table II). Five cancers were clinically confined to the prostate (one T1 and four T2) and were treated by radical prostatectomy. Pathological staging confirmed organ-confined disease in four cases and revealed seminal vesicle invasion in one of the clinically localised tumours. The exact pathological stage for these cases were: T1b, T2a, T2a, T2c, T3c. One case had both local invasion and bone metastases, (clinical stage T3 Nx M1) and was treated with endocrine therapy (LHRH agonist). No pathological staging was available for this case.

All men with elevated PSA (4.0 $\mu g\ l^{-1}$ or above), but without cancer diagnosis in the initial examination, were re-examined with DRE and biopsy at 1 year and those with PSA 10 $\mu g\ l^{-1}$ also at 6 months from the initial examination. The follow-up has not revealed any additional prostate cancers.

The ratio of free to total PSA (F/T PSA) was also measured for all men. The relationship of the serum total PSA concentration and F/T PSA is shown in Figure 2. Among men with serum PSA concentration of 4.0 $\mu g\ l^{-1}$ or above, the use of F/T PSA as an additional criterion

**Figure 1** The serum total PSA concentration ($\mu g l^{-1}$) by age among men with and without prostate cancer, Finnish pilot study of prostate cancer screening. ●, Prostate cancer; ○, no cancer.

**Table I** Results of the pilot PSA screening study in Finland

| Age | Invited (N) | Participated (%) | 2⩽PSA <4 (%) | PSA ⩾4 (%) | Cancer (%) | PPV (%) | $pT_{1-2}$ |
|---|---|---|---|---|---|---|---|
| 55 | 100 | 78 | 8 | 5 | 2.6 | 50 | 1/2 |
| 60 | 100 | 64 | 7 | 13 | 1.6 | 13 | 1/1 |
| 65 | 100 | 90 | 17 | 14 | 3.3 | 23 | 2/3 |
| Total | 300 | 77 | 11 | 11 | 2.6 | 24 | 4/6 |

PPV, positive predictive value.

**Table II** Characteristics of the cancers detected in the Finnish pilot study of prostate cancer screening

| Case no. | Age | PSA | F/T PSA | DRE | TRUS | Grade | Stage |
|---|---|---|---|---|---|---|---|
| 1 | 65 | 26.9 | 0.08 | Suspect | Normal | I | pT2cN0M0 |
| 2 | 65 | 26.0 | 0.07 | Suspect | Normal | II | cT3NxM1 |
| 3 | 55 | 9.6 | 0.06 | Suspect | Normal | II | pT3cN0M0 |
| 4 | 60 | 6.3 | 0.31 | Suspect | Normal | I | pT1bN0M0 |
| 5 | 55 | 5.5 | 0.17 | Suspect | Normal | I | pT2aN0M0 |
| 6 | 65 | 5.3 | 0.17 | Suspect | Suspect | I | pT2aN0M0 |

**Figure 2** The total serum PSA concentration ($\mu g\, l^{-1}$) by the proportion of free to total PSA among men with and without prostate cancer, Finnish pilot study of prostate cancer screening. ○, No cancer; ●, prostate cancer; +, not biopsied.

increased the positive predictive value of the screening test. When the cut-off level of 0.20 for F/T PSA was used (with decreased values indicating a high risk of prostate cancer), the positive predictive value for the combination of the two tests was 42%. This procedure would have resulted in a reduction of screening positive findings by half to 12 men (5%), i.e. a reduction in false-positive screening tests from 8% to 3%. This would have been obtained at a cost of missing one localised prostate cancer.

No cases of prostate cancer have yet been diagnosed in the control group or among non-participants (2 years from randomization).

### Discussion

Our study was population based, which, combined with a high participation rate (77%), efficiently improved external validity and reduced selection bias compared with self-enrolment used in most other studies. Nevertheless, owing to the small number of subjects in our feasibility study, one should be cautious in drawing conclusions. The high participation rate observed in our study indicates that screening based on serum PSA concentration is feasible in Finland and that Finnish men aged 55–65 years regard the screening procedure as acceptable. However, we have not at this stage approached participants and non-participants to evaluate satisfaction and reasons for non-participation.

A relatively low cut-off level for PSA concentration was chosen to allow assessment of higher thresholds for screening. Four out of six men with prostate cancer had a PSA concentration below 10 $\mu g\, l^{-1}$, suggesting that most of the cancers would be missed, at least in a single screening round, if a higher cut-off level were to be used. We used the Delfia kit by Wallac for determining the total PSA concentration. It has a very good correlation with, e.g. the Hybritech Tandem-R method ($r=0.99$) (Stenman *et al.*, 1990).

The proportion of screening-positive men (11%) was similar to that observed in other studies (Chadwick *et al.*, 1991; Brawer *et al.*, 1992; Labrie *et al.*, 1992; Catalona *et al.*, 1993; Mettlin *et al.*, 1993*b*). This implies that PSA-based screening requires a relatively large number of urological examinations at a considerable cost. It may be possible to improve the positive predictive value of the PSA test, and thus to reduce the need for diagnostic examinations, by determination of the free and total PSA (Stenman *et al.*, 1994). Other modifications to the PSA test, such as PSA density, PSA velocity or age-referenced PSA, do not seem to improve the performance of the screening test compared with serum PSA concentration alone (Catalona *et al.*, 1994; Mettlin *et al.*, 1994).

The positive predictive value of 24% in our study can be considered fairly good, although it indicates that three out of four screening-positive men are actually free of the disease. The most important source of false-positive tests seems to be prostatic hyperplasia. It might by improved by applying a higher cut-off level, but this would automatically diminish sensitivity.

These figures can be compared with those observed in breast cancer screening. In mammography screening for breast cancer, 4% of women have a screening positive finding and are subject to further diagnostic examination (Hakama *et al.*, 1991). Breast cancer is diagnosed in 0.4% of the screened women, which corresponds to a positive predictive value of 10% (Hakama *et al.*, 1991).

One must bear in mind, however, that the results are obtained from the first screening round in a previously unscreened population. In a prevalence screen, the yield may be higher and the tumours less aggressive than in subsequent screening rounds. This may also affect the performance characteristics of the screening test, including sensitivity, specificity and stage distribution.

In our study, sensitivity and specificity could not be assessed because the true prevalence of disease was not known among men with PSA below 4.0 $\mu g\, l^{-1}$. The same applies to the negative predictive value. In the literature, a sensitivity of 72%, specificity of 91% and negative predictive value of 98% have been reported for a cut-off level of 4.0 $\mu g\, l^{-1}$ in a study with DRE, TRUS and PSA as independent indications for biopsy (Labrie *et al.*, 1992).

One of the potential problems in screening for prostate cancer is the possibility of overdiagnosis, i.e. screening may lead to detection of indolent lesions that may fulfil the histological criteria of cancer, but would never surface clinically during the subject's lifetime. The detection rate in our study (2.6%) was similar to earlier studies, in which it has ranged from 1% to 5% (Chadwick *et al.*, 1991; Brawer *et al.*, 1992; Labrie *et al.*, 1992; Catalona *et al.*, 1993). However, it was quite high relative to the life-time risk of developing a prostate cancer. Based on the age-specific incidence rates in Finland, the detection rate of 2.6% corresponds to the expected number of prostate cancers within 10 years of follow-up of the screened men (assuming constant incidence rates at the level of 1992 for the whole of Finland). In Finland, the life-time risk of prostate cancer between the ages 55 and 80 is approximately 9%. Comparison of this figure with the yield of 2.6% in our study suggests that to avoid overdiagnosis, a single screening round should reduce the life-time risk of subsequent prostate cancer by at least 25%. The first results suggest, however, that the detection rate in the following screening rounds is probably 25–50% smaller compared with the prevalence screen (Catalona *et al.*, 1993; Mettlin *et al.*, 1993*b*). Thus, the probability of overdiagnosis seems to decrease in the consecutive screening rounds. The situation may be similar to that encountered in cervical cancer screening, where all invasive *in situ* lesions are treated, although only a quarter of them would develop into cancer (Hakama and Räsänen-Virtanen, 1976).

Several case–control studies based on serum banks have been published that enable retrospective assessment of PSA level preceding the diagnosis of prostate cancer (Carter *et al.*, 1992; Helzlsouer *et al.*, 1992; Paus *et al.*, 1992; Stenman *et al.*, 1994; Gann *et al.*, 1995; Whittemore *et al.*, 1995). In these studies, no screening procedures were performed apart from drawing a blood sample for storage. Later on, the samples were analysed from subjects with subsequent prostate cancer and from controls. Thus, the advantage of these studies is the lack of overdiagnosis, i.e. over-representation of lesions that would never have developed into a symptomatic phase. The results suggest that serum PSA has a high sensitivity up to 5 years before clinical surfacing of prostate cancer and an elevation of PSA can be detected up to 10 years before diagnosis. Thus, the sensitivity of the test may after all be good and overdiagnosis avoidable.

A prerequisite for screening is detection of cases at curable stage. In our study, five of the six cancers detected had clinically local stage and pathological staging confirmed that the disease was confined to the prostate in four cases. This is a more favourable stage distribution than that observed among cases diagnosed through normal clinical practice in the same ages (according to data from the Finnish Cancer Registry, approximately 45% of prostate cancers in Finland are currently diagnosed at local stage). This finding is in accordance with previous reports (Catalona *et al.*, 1993; Mettlin *et al.*, 1993*a*). Only one of the six tumours was small (T1b), suggesting that the screening-detected cancers are not indolent, but have clinical relevance.

Our results, although based on a small sample, support earlier results, suggesting that the combination of ratio of free to total PSA increases the specificity and the positive predicitive value of the screening test (Stenman *et al.*, 1994). The combination of the two tests, the total PSA and the ratio of free to total PSA, has the potential to substantially reduce the number of false-positive findings and hence the costs and harmful side-effects of a screening programme. However, the improved sensitivity can only be gained with some loss of specificity. In our study, one of the six cancers would have been missed if the screening positive finding was defined as serum PSA concentration of 4.0 $\mu g\ l^{-1}$ or higher and F/T PSA 0.20 or below. We find this level of false negatives is acceptable, especially as overdiagnosis is one of the most important concerns for prostate cancer screening. Nevertheless, the usefulness of the F/T PSA will only be determined with further experience as our results need to be confirmed in larger studies. Also, more studies on the optimal cut-off level of the F/T PSA are needed.

Evaluation of the efficacy of a screening programme should be based on reduction of mortality from the disease, which can be done only on the basis of large randomised studies (Miller, 1982). In prostate cancer, the potential reduction of prostate cancer mortality remains to be demonstrated with long-term follow-up and it may be small. Because of this and the facts that the treatment is associated with high frequency of side-effects and that the saved years of life are at an older age, assessment of quality of life is essential for evaluation of the possible benefit and harm caused by prostate cancer screening. Furthermore, the cost-effectiveness of the screening programme should be assessed. So far, no data are available regarding the effect of prostate cancer screening on mortality from the disease, nor on quality of life or cost-efficiency. A European collaborative study has been set up to investigate these end points, with Finland as a participating country (Schröder *et al.*, 1995).

The results of our pilot study using a serum PSA cut-off level of 4.0 $\mu g\ l^{-1}$ suggest that a high participation rate can be achieved in the general population and that the proportion of screening-positive men, positive predictive value and detection rate are at an acceptable level. Thus, a population-based randomised study on screening for prostate cancer with prostate cancer mortality, quality of life and cost-efficiency as end points is feasible in Finland.

**Acknowledgements**
The authors thank the Mr Esko Voutilainen at the Finnish Cancer Registry as well as Ms Emmi Vettenranta and Ms Seija Katavisto at the Pirkanmaa Cancer Society clinic for valuable assistance. We are also grateful to Barry Dowell from Abbott for kindly providing the monoclonal antibodies H117 and H50.

### References

BRAWER MK, CHETNER MP, BEATIE J, BUCHNER DM, VESSELLA RL AND LANGE PH. (1992). Screening for prostatic carcinoma with prostate specific antigen. *J. Urol.*, **147,** 841–845.

CARTER HB, PEARSON JD, METTER J, BRANT LJ, CHAN DW, ANDRES R, FOZARD JL AND WALSH PC. (1992). Longitudinal evaluation of prostate-specific antigen levels in men with and without prostate cancer. *J. Am. Med. Assoc.*, **267,** 2215–2220.

CATALONA WJ, SMITH DS, RATLIFF TL AND BASLER JW. (1993). Detection of organ-confined prostate cancer is increased through prostate-specific antigen-based screening. *J. Am. Med. Assoc.*, **270,** 948–954.

CATALONA WJ, RICHIE JP, DEKERNION JB, AHMANN FR, RATLIFF TL, DALKIN BL, KAVOUSSI LR, MACFARLANE MT AND SOUTHWICK PC. (1994). Comparison of prostate specific antigen concentration versus prostate specific antigen density in the early detection of prostate cancer: receiver operator curves. *J. Urol.*, **152,** 2031–2036.

CHADWICK DJ, KEMPLE T, ASTLEY JP, MACIVER AG, GILLATT DA, ABRAMS P AND GINGELL JC. (1991). Pilot study of screening for prostate cancer in general practice. *Lancet*, **338,** 613–616.

COLEMAN MP, ESTEVE J, DAMIECKI P, ARSLAN A AND RENARD H. (1993). *Trends in Cancer Incidence and Mortality.* IARC Scientific Publications No. 121. International Agency for Research on Cancer: Lyon.

GANN PH, HENNEKENS CH AND STAMPFER MJ. (1995). A prospective evaluation of plasma prostate-specific antigen for detection of prostatic cancer. *J. Am. Med. Assoc.*, **273,** 289–294.

HAKAMA M, ELOVAINIO L AND LOUHIVUORI K. (1991). Breast cancer screening programme in Finland. *Br. J. Cancer*, **64,** 962–964.

HAKAMA M AND RÄSÄNEN-VIRTANEN U. (1976). Effect of a mass screening program on the risk of cervical cancer. *Am. J. Epidemiol.*, **103,** 512–517.

HELZLSOUER KJ, NEWBY J AND COMSTOCK GW. (1992). Prostate-specific antigen levels and subsequent prostate cancer: potential for screening. *Cancer Epidemiol. Biomarkers Prev.*, **1,** 537–540.

LABRIE F, DUPONT A, SUBURU R, CUSAN L, TREMBLAY M, GOMEZ J-L AND EMOND J. (1992). Serum prostate specific antigen as pre-screening test for prostate cancer. *J. Urol.*, **147,** 846–852.

LEINONEN J, ZHANG W-M AND STENMAN U.-H. (1996). Complex formation between PSA isoenzymes and protease inhibitors. *J. Urol.*, **155,** 1099–1103.

METTLIN C, MURPHY GP, LEE F, LITTRUP PJ, CHESLEY A, BABAIAN R, BADALAMENT R, KANE RA AND MOSTOFI FK. (1993*a*). Characteristics of prostate cancer detected in a multimodality early detection program. *Cancer*, **72,** 1701–1708.

METTLIN C, MURPHY GP, RAY P, SHANBERG A, TOI A, CHESLEY A, BABAIAN R, BADALAMENT R, KANE RA, LEE F AND THE INVESTIGATORS OF THE AMERICAN CANCER SOCIETY NATIONAL PROSTATE CANCER DETECTION PROJECT. (1993*b*). American Cancer Society National Prostate Cancer Detection Project. Results from multiple examinations using transrectal ultrasound, digital rectal examination and prostate specific antigen. *Cancer*, **71,** 891–898.

METTLIN C, LITTRUP PJ, KANE RA, MURPHY GP, LEE F, CHESLEY A, BADALAMENT R AND MOSTOFI FK FOR THE INVESTIGATORS OF THE AMERICAN CANCER SOCIETY NATIONAL PROSTATE CANCER DETECTION PROJECT. (1994). Relative sensitivity and specificity of serum prostate specific antigen (PSA) level compared with age-referenced PSA, PSA density and PSA change. *Cancer*, **74,** 1615–1620.

MILLER AB. (1982). Fundamental issues in screening. In: *Cancer Epidemiology and Preventions.* Schottenfeld D and Fraumeni JF JR. (eds). WB Saunders: Philadelphia, pp. 1064–1074.

PAUS E, THEODORSEN L AND ENGELAND A. (1992). Prostate-specific antigen in serum from blood donors with subsequent prostate cancer diagnosis. *Eur. J. Cancer*, **29A,** 1221–1222.

SCHRÖDER FH, DENIS LJ, KIRKELS W, DE KONING HJ AND STANDAERT B. (1995). European randomized study of screening for prostate cancer. *Cancer*, **76,** 129–134.

STENMAN U-H, BJÖRSES U-M AND LEINONEN J. (1990). Time-resolved immunofluorometry assay of prostate-specific antigen. *J. Nucl. Med. All. Sci.*, **34** (suppl. 3), 249–251.

STENMAN U-H, HAKAMA M, KNEKT P, AROMAA A, TEPPO L AND LEINONEN J. (1994). Serum concentrations of prostate specific antigen and its complex with $\alpha_1$-antichymotrypsin before diagnosis of prostate cancer. *Lancet*, **344**, 1594–1598.

TEPPO L, PUKKALA E AND LEHTONEN M. (1994). Data quality and quality control of a population-based cancer registry. *Acta Oncol.*, **33**, 365–369.

WALSH PC, PARTIN AW AND EPSTEIN JI. (1994). Cancer control and quality of life following anatomical radical retropubic prostatectomy. *J. Urol.*, **152**, 1831–1836.

WHITTEMORE AS, LELE C, FRIEDMAN GD, STAMEY T, VOGELMAN JH AND ORENTREIH N. (1995). Prostate-specific antigen as predictor of prostate cancer in black men and white men. *J. Natl Cancer Inst.*, **87**, 354–360.

WILSON JMG AND JUNGNER G. (1969). *Principles and Practice of Screening for Disease*. Public health paper No. 34. World Health Organization: Geneva.

ZAGARS GK, POLLACK A, KAVADI VS AND VON ESCHENBACH AC. (1995). Prostate-specific antigen and radiation therapy for clinically localized prostate cancer. *Int. J. Radiat. Biol. Oncol. Phys.*, **32**, 293–306.